# Diagnostic accuracy of computed tomography imaging for the detection of differences between peripheral small cell lung cancer and peripheral non-small cell lung cancer

**DOI:** 10.1007/s10147-017-1131-0

**Published:** 2017-05-09

**Authors:** Yanchen Ren, Yiyuan Cao, Weidong Hu, Xiaoxuan Wei, Xiaoyan Shen

**Affiliations:** 1 Department of Thoracic Surgical Oncology, Hubei Key Laboratory of Tumor Biological Behaviors and Hubei Cancer Clinical Study Center, Zhongnan Hospital of Wuhan University, 430071 Wuhan, People’s Republic of China; 2 Department of Radiology, Medical Imaging Center, Zhongnan Hospital of Wuhan University, 430071 Wuhan, People’s Republic of China

**Keywords:** CT features, Peripheral small cell lung cancer, Peripheral non-small cell lung cancer, Sensitivity, Specificity

## Abstract

**Background:**

To evaluate the computed tomography features of peripheral small cell lung cancer and non-small cell lung cancer and to establish a predictive model to conveniently distinguish between them.

**Materials and methods:**

We retrospectively reviewed the computed tomography features of 51 patients with peripheral small cell lung cancer and 207 patients with peripheral non-small cell lung cancer after pathological diagnosis. Thirteen computed tomography morphologic findings were included and analyzed statistically. Meaningful features were analyzed by logistic regression for multivariate analysis. We then used *β*-coefficients as the basis to establish an image scoring prediction model.

**Result:**

The meaningful morphologic features for distinguishing between peripheral small cell lung cancer and other tumor types are multinodular shape and lymphadenectasis, with scores of 12 and 11, respectively. The scores ranged from −51 to 23, and the most reasonable cut-off was −24. The available area under the curve was 0.834 (95% confidence interval [CI] 0.783–0.877). Sensitivity and specificity were 86.3% (95% CI 0.737–0.943) and 69.6% (95% CI 0.628–0.758), respectively.

**Conclusion:**

The image scoring predictive model that we constructed provides a simple and economical noninvasive method for distinguishing between peripheral small cell lung cancer and peripheral non-small cell lung cancer.

## Introduction

Lung cancer is a malignant tumor with high morbidity and mortality and is the leading cause of cancer death worldwide [1]. In China, there is a similar situation regarding lung cancer, with official statistics showing that of 10 common cancers, lung cancer has the highest morbidity and mortality rates [2]. Depending on the different locations of the lesion, lung cancer can be divided into central type and peripheral type. According to the different biological characteristics, lung cancer can also be divided into small cell lung cancer (SCLC) and non-small cell lung cancer (NSCLC) using the World Health Ognization (WHO) pathological staging system [3].

The central type of lung cancer refers to tumors that occur above the lung segment bronchus, and the peripheral type of lung cancer refers to tumors that occur below the lung segment bronchus. Peripheral lung cancer accounts for >70% of all lung cancers [4]. As a specific clinical and histological lung cancer, SCLC accounts for approximately 15–20% of all lung cancers [5]; it is characterized by short doubling time, early lymph node metastasis and distant metastasis. The incident rate of distant metastases is approximately 60–70% in those patients who were initially diagnosed as SCLC [6]. Although the annual incidence of SCLC in industrialized countries has decreased over the past 30 years, the incidence of SCLC has increased in countries where smoking prevalence remains high, such as Eastern Europe and Asia [7]. A multicenter epidemiological study conducted in China shows that the incidence of SCLC in all lung cancers increased from 13.7 to 18.3% between 2005 and 2010 [8].

In SCLC, the rate of central type is approximately 70–85%. Generally, central type is not considered for surgical treatment. In contrast, the rate of peripheral type is approximately 15–30%. For these patients, surgical treatment may be useful to improve overall survival. Only 4–12% of tumors present as solitary nodules in all SCLCs [9], and for patients whose tumor is diagnosed as stage I (T1-2N0M0), there is a possibility to undergo surgery with curative intent [10]. For NSCLC, if the patient is strongly clinically suspected of having stage I or II cancer, a histological test is not required before surgery [11]. However, for peripheral lung cancer patients, the treatment for NSCLC and SCLC may be completely different. The operation is likely to be the best choice for early stage NSCLC, but unlike NSCLC, most SCLC patients cannot benefit from surgery [12]. It is a problem for clinicians to identify between these two diseases, and there is a necessity for a convenient and accurate diagnosis method.

The most inexpensive and effective noninvasive diagnostic facility for lung cancer, i.e., the CT scanner, has been installed in most hospitals in China. Although there are many studies on the CT features of peripheral SCLC (PSCLC) [4, 6, 9, 13–15], there is few case−control study investigating the differences between PSCLC and peripheral NSCLC (PNSCLC) images. To date, there is still no easy way to distinguish between PSCLC from PNSCLC on CT images. In this study, we will introduce a simple and effective diagnostic prediction model based on CT features for distinguishing between PSCLC and PNSCLC.

## Materials and methods

### Study population

The retrospective database was from the Zhongnan Hospital of Wuhan University. All patients provided signed informed consent before undergoing the CT examination. We selected PSCLC and PNSCLC cases that were confirmed by pathological diagnosis at Zhongnan Hospital of Wuhan University from January 2004 to September 2014. Inclusion criteria were (a) preoperative and continuous CT scans of the chest had been performed, and (b) CT scans of the abdomen, bone scan, and magnetic resonance imaging (MRI) or CT scans of the brain had been performed to diagnose tumor metastasis. Exclusion criteria were (a) central type tumors detected by CT, and (b) patients who were previously diagnosed with other cancers.

### Acquisition of CT images

All CT features were performed with CT scanners (Somatom Sensation16 and Somatom Definition 64; Siemens Healthcare, Forchheim, Germany) in helical mode from the apex to the lung base. The patient was positioned in the supine position. Technical parameters were X-ray tube current 100 mA; tube voltage 120 kV; collimation 5 mm; rotation speed 0.5 s; matrix 512 × 512. All image data were interfaced directly to our picture archiving and communication system. Monitors were used to view both mediastinal and lung window images.

### CT image analysis

**Fig. 1:**
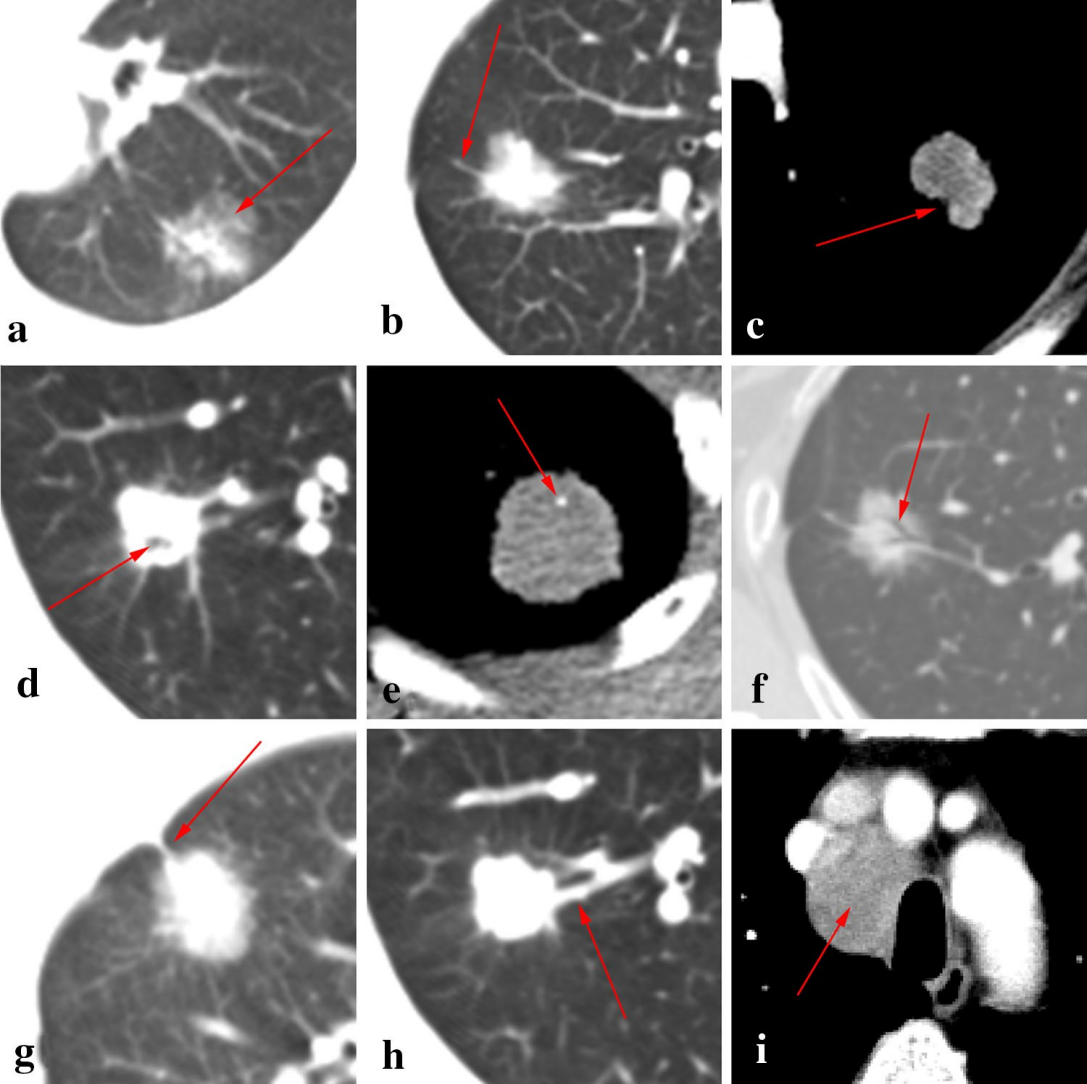
CT features. **a** The halo sign is a CT finding of ground-glass opacity surrounding a nodule or mass. **b** The spiculated sign is when the edge of a nodule or mass extends to the surrounding lung parenchyma, which contains linear strands that extend into the tissue of the lung but not into the pleural margin. **c** The lobulation sign is when the edge of the nodule shows uneven lobulated contour. **d** The cavity sign is a gas-filled space, seen as a lucency or low-attenuation area, within a pulmonary consolidation, mass, or nodule. **e** The calcification showed high attenuation, the average CT value of non-enhanced CT is >100 Hu. **f** The air bronchogram is a pattern of air-filled (low-attenuation) bronchi on a background of an opaque (high-attenuation) airless lung. **g** Pleural indentation is showed as a tapered or linear extension of the lesion to pleura, reflect the pulmonary fibrosis with adjacent pleural retraction. **h** The bronchovascular convergence sign is when one or more vessels reach the edge of the tumor or cross the tumor. **i** Lymphadenectasis means the size of a mediastinal or hilar lymph node is >1 cm in *short axis* diameter

Descriptions of imaging features conform to the Nomenclature Committee of the Fleischner Society (NCFS) chest image definition [16]. All CT images were reviewed by the same two senior radiologists, who assessed and recorded lesion size and shape, single/multifocal lesion, halo sign, lobulation sign, spiculated sign, calcification, cavity, air bronchogram, the bronchovascular convergence sign, pleural indentation, pleural effusion, lymphadenectasis, distant metastasis and other signs (see Fig. 1 for definitions).

### Reference standard

Peripheral lung cancer in this study was determined using the pathological results from various pathological biopsies. All the specimens were performed by immunohistochemistry and diagnosed as SCLC or NSCLC.

### Statistical analysis

To evaluate the predictive factors for the pathological types of peripheral lung cancers, statistical differences were analyzed using the Mann–Whitney Wilcoxon test for continuous data and chi-squared test for categorical data, using a significance level of *P* = 0.05. To identify significant predictors of CT features in PSCLC, multivariate logistic regression analysis was conducted (forward LR method). The removal of variables was based on likelihood ratio statistics with a probability of 0.10. The regression coefficients of significant CT features which were detected by multivariate logistic regression analysis were regarded as independent variable factors, multiplied by ten and rounding to the nearest integer. We calculated all significant features together in every peripheral lung cancer patient using these numbers as scores. We then took the sum of the scores to draw receiver operating characteristic (ROC) curves and calculated the available area under the curve (AUC). We used the Youden index to calculate the best cut-off.

## Results

Between January 2004 and September 2015 (the date of patients diagnosed with non-small cell lung cancer ranged from September 2014 to September 2015), 655 patients were diagnosed with lung cancer. The screening criterion was untreated primary lung cancer patients with completed CT and pathological data. There were 220 cases of SCLC (169 cases of central type, 51 cases of peripheral type) and 435 cases of NSCLC (226 cases of central type, 207 cases of peripheral type). In 207 cases of PNSCLC, there were 48 squamous cell carcinomas, 139 adenocarcinomas, seven low differentiated carcinomas, five sarcomatoid carcinomas, one neuroendocrine carcinoma (non-small cell), three adenosquamous carcinomas and four undifferentiated non-small cell lung cancer.

According to the TNM for the 7^th^ edition of the AJCC staging system, there were 11 cases of stage I, 10 cases of stage II, 10 cases of stage III and 20 cases of stage IV in 51 cases of PSCLC; there were 59 cases of stage I, 29 cases of stage II, 31 cases of stage III and 88 cases of stage IV in 207 cases of PNSCLC.

### CT features and scoring

Table 1 shows the CT features of all patients who were diagnosed with peripheral lung cancer. We analyzed the size of peripheral lung nodules and characteristic CT features. By measuring and comparing the size of the lesions, it was found that the size of peripheral lung cancer lesions did not conform to normal distribution—the median was 40 mm in PSCLC, and 37 mm in PNSCLC (*P* > 0.05), meaning that there was no difference between PSCLC and PNSCLC in lesion size. Statistical analysis showed that six CT features were significant in the diagnosis (*P* < 0.01) (Table 1).

**Table 1:** Comparison of the CT features between PSCLC and PNSCLC

CT feature	PSCLC (%)	PNSCLC (%)	Chi-squared	*P*
Shape*			18.46	<0.001
Round/ovoid shape	30 (58.8)	113 (54.6)		
Irregular shape	7 (13.7)	76 (36.7)		
Multinodular shape	14 (27.5)	18 (8.7)		
Single lesion	44 (86.3)	188 (90.8)	0.933	0.334
Halo sign	5 (9.80)	13 (6.3)	0.334	0.563
Spiculated sign*	28 (54.9)	178 (86.0)	24.575	0.000
Lobulation sign	42 (82.4)	172 (83.1)	0.016	0.900
Cavity*	21 (41.2)	149 (72.0)	17.276	0.000
Calcification	10 (19.6)	28 (13.5)	1.205	0.272
A|ir bronchogram	5 (9.8)	36 (17.4)	1.762	0.184
Pleural indentation*	19 (37.3)	139 (67.1)	15.406	0.000
Bronchovascular convergence sign*	26 (51.0)	128 (81.5)	18.689	0.000
Pleural effusion	5 (9.8)	19 (9.2)	0.000	1
Lymphadenectasis*	31 (60.8)	82 (39.6)	7.451	0.006
Distant metastasis	20 (39.2)	88 (42.5)	0.183	0.669
Total	51	207		

* Differences were statistically significant

**Fig. 2:**
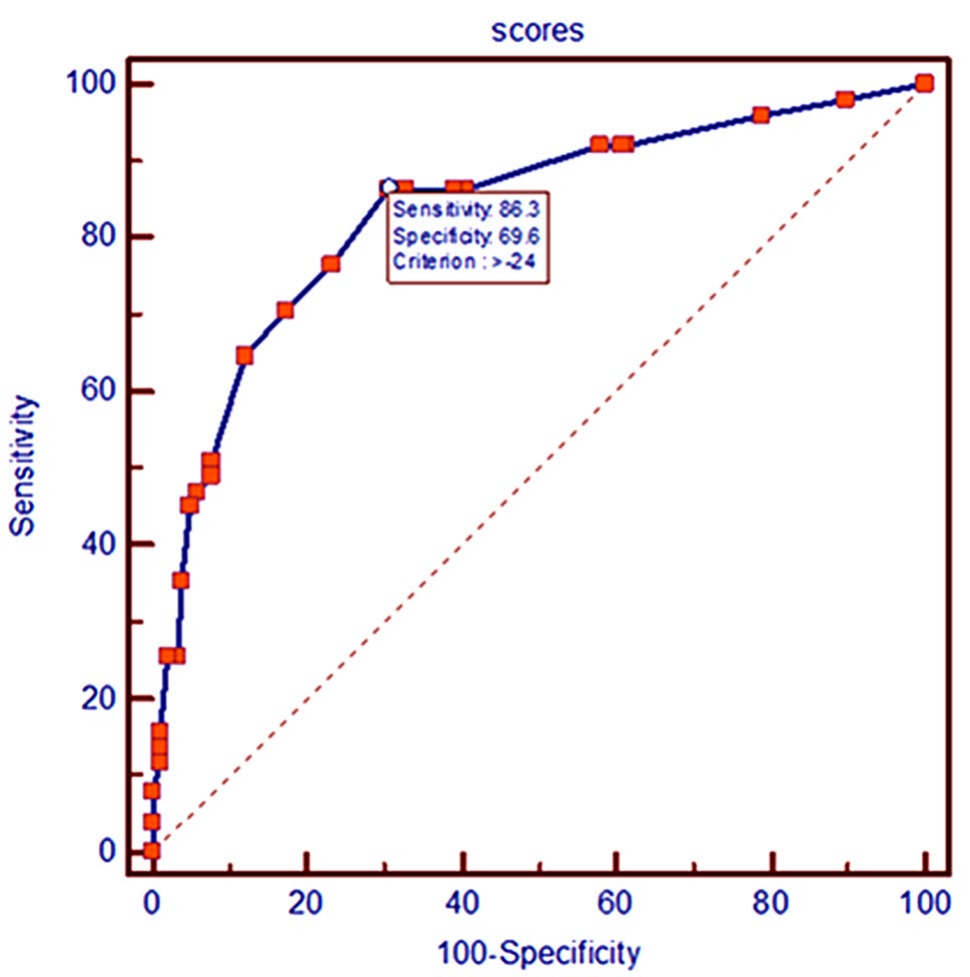
ROC of radiographic scores of peripheral lung cancer on pathological types

Multivariate logistic regression analysis was performed to identify the diagnosis effect of the 6 CT features (shape, spiculated sign, cavity, pleural indentation, bronchovascular convergence sign and lymphadenectasis) with statistical significance. It had a tendency toward PSCLC diagnosis with the CT features of lymphadenectasis and multinodular shape (*P* < 0.05); however, irregular shape, spiculated sign, cavity and pleural indentation sign had a higher tendency toward PNSCLC diagnosis (*P* < 0.05). In order to facilitate the clinical application, we set up a mathematical model based on the results of two logistic regression analyses, multiplied the *β*-coefficients of the significant CT feature by 10 and rounded to the nearest integer for analysis (Table 2). The scores of each CT feature were added together to get the final score of each patient, and we then obtained a group of continuous variables with a score of −51 to 23. The final score was chosen as variable, and the pathological diagnosis as the dependent variable (1 = PSCLC, 0 = PNSCLC), using the statistical software MedCalc to draw the ROC curve (Fig. 2); the AUC was 0.834 (95%CI 0.783–0.877). The maximum value of the Youden index was 0.5584, which corresponded to the most reasonable cut-off of −24. When the final score was more than −24, the diagnosis was PSCLC, and the sensitivity and specificity were 86.3% (95%CI 0.737−0.943) and 69.6% (95%CI 0.628−0.758), respectively.

**Table 2:** The image scoring prediction model of CT features analysis based on logistic regression for diagnosis of peripheral lung cancer

Variable	*β*-Coefficients	*P* value	OR (95% CI)	Score
Multinodular shape	1.208	0.012	3.345 (1.310–8.546)	12
Irregular shape	−1.256	0.012	0.285 (0.107–0.758)	−13
Spiculated sign	−1.386	0.001	0.250 (0.114–0.551)	−14
Cavity	−1.289	0.001	0.275 (0.132–0.576)	−13
Pleural indentation	−1.107	0.003	0.331 (0.159–0.690)	−11
Lymphadenectasis	1.081	0.005	2.947 (1.382–6.287)	11

## Discussion

Since many surgeons perform surgery without invasive examinations, CT inspection plays an important role in the diagnosis and treatment of SCLC. Although it has been stated that peripherally located SCLC often shows different CT features from other lung cancers [15], there is no unified standard model to evaluate if a CT feature is related to PSCLC. In such an environment, the image scoring prediction model that we have invented can distinguish PSCLC from PNSCLC easier than other noninvasive methods.

PSCLC tumors are always described as having homogeneous density, with rounded lobulated nodules and smooth edges in CT images, with little spiculated sign, pleural indentation and internal necrosis [13]. Many articles have studied the CT features of PSCLC and drawn some conclusions [4, 6, 9, 13–15, 17–19], however, their conclusions were varied because of the lack of uniform CT features and the small number of cases and case−control studies for PNSCLC. Most important, however, the conclusions they made were subjective and lacked generality and accuracy. Nevertheless, they also provided many clues from which we were able to draw conclusions to use as a foundation. We then improved the evaluation method and invented a radiological score system to make the diagnosis accurate, simple and practical.

We established a simple and practical mathematical model for CT imaging to distinguish between PSCLC and PNSCLC. This model is based on the pathological type of peripheral lung cancer. The scoring options of the system comprise five CT features—shape, spiculated sign, cavity, pleural indentation and lymphadenectasis, which were selected from thirteen common CT features of peripheral lung cancer. It had a tendency toward PSCLC diagnosis with the CT features of multinodular shape and lymphadenectasis; however, irregular shape, spiculated sign, cavity and pleural indentation sign had a higher tendency toward PNSCLC diagnosis.

**Fig. 3:**
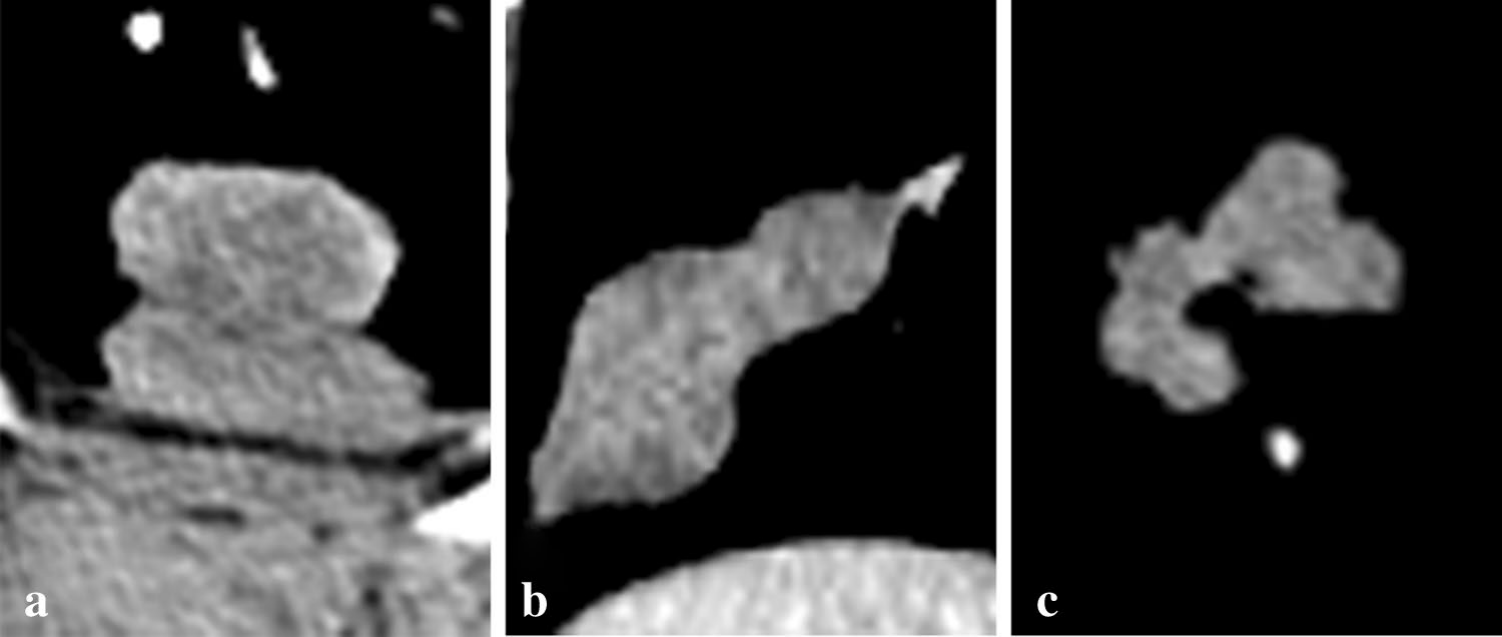
The shape of multinodular tumors

In our study, the shape of the peripheral pulmonary nodules had important implications for diagnosing the pathology of tumors. According to the CT, there are three shape characteristics for PSCLC tumors—round/ovoid shape, irregular shape and multinodular shape. Although the round/ovoid shape might be the most common type of PSCLC [15, 20], it is also a common type of PNSCLC and therefore cannot be used as a feature to distinguish between these two diseases. The multinodular shape, defined as spindle-like and whose major axis points to the hilum, performs as a polymer that is aggregated by two or more nodules (Fig. 3), and is characteristic of PSCLC. In previous literature, the multinodular shape was often described as fusiform, vermiform, beaded, etc. [5, 15]. The multinodular shape might be related to the growth pattern of tumors that grow along the bronchial/blood vessel wall with short doubling time. Under these circumstances, the tumor is oppressed by the surrounding tissues, and it grows unbalanced. When the tumor grows surrounding bronchioles, it manifests as adjacent multiple nodules in the axial, which may be the reason why PSCLC is multinodular.

Lymphadenectasis is also reported as a significant CT feature of PSCLC. For histological consideration, the boundary of SCLC is not clear, as it grows as a cluster or nest aggregation pattern, with lack of fibrous tissue inside [3]. The tumor grows along the small bronchial wall or the bronchial artery and can easily invade the lung parenchyma; this expression may be related to local progression of the tumor, including hilar and mediastinal lymph node metastasis [15].

In our study, pleural indentation is a feature that can be diagnosed as being characteristic of PNSCLC. The reason can be revealed by histologic examination which described that the tumor cells of SCLC tend to grow and form clusters or nests in the peripheral alveoli. This conclusion was similar to the results of Nobukata et al. [20], and this may be the reason why cavity formation had a tendency toward PNSCLC diagnosis.

An important finding in our study is that TNM staging is not a feature for distinguishing between PSCLC and PNSCLC; this is another one of the reasons why PSCLC is hard to detect from peripheral lung cancer. This finding also gives indirect evidence to the importance of our image scoring system model.

The image scoring system model converted the CT features of peripheral lung cancer to qualitative data through logistic regression and ROC curve analysis (Table 2). When the final score was more than −24, the diagnosis was PSCLC, and the sensitivity and specificity were 86.3 and 69.6%, respectively. The AUC was 0.834, indicating that the accuracy of the diagnosis was moderate.

There are several limitations in our model. First, as a retrospective design, some bias may exist in our study. Second, the decision on whether patients had distant metastasis was based on the results of clinical and imaging studies instead of pathological investigation. Third, some CT features, such as nodules <1 cm in both PSCLC and PNSCLC, were not typical CT findings. All of these factors might lead to bias in diagnosis.

In conclusion, we established a scoring system using the CT features of peripheral lung cancer, which provided a basis for clinical treatment selection, and was also a simple and economical method for the noninvasive diagnosis of lung cancer patients.

## Abbreviations

SCLC: Small cell lung cancer
NSCLC: Non-small cell lung cancer
PSCLC: Peripheral small cell lung cancer
PNSCLC: Peripheral non-small cell lung cancer
95% CI: 95% Confidence interval
CT: Computed tomography
MRI: Magnetic resonance imaging
PET: Positron emission tomography
NCFS: Nomenclature Committee of the Fleischner Society
ROC: Receiver operating characteristics curves
AUC: Available area under the curve
NCCN: National Comprehensive Cancer Network
